# Direct Observation of Compartment-Specific Localization and Dynamics of Voltage-Gated Sodium Channels

**DOI:** 10.1523/JNEUROSCI.0086-22.2022

**Published:** 2022-07-13

**Authors:** Hui Liu, Hong-Gang Wang, Geoffrey Pitt, Zhe Liu

**Affiliations:** ^1^Janelia Research Campus, Howard Hughes Medical Institute, Ashburn, Virginia 20147; ^2^Weill Cornell Medicine, Cardiovascular Research Institute, New York, New York 10021

**Keywords:** genome editing, protein transport and trafficking, single-molecule imaging, subcellular localization, voltage-gated sodium channel

## Abstract

Brain enriched voltage-gated sodium channel (VGSC) Na_v_1.2 and Na_v_1.6 are critical for electrical signaling in the CNS. Previous studies have extensively characterized cell-type-specific expression and electrophysiological properties of these two VGSCs and how their differences contribute to fine-tuning of neuronal excitability. However, because of a lack of reliable labeling and imaging methods, the subcellular localization and dynamics of these homologous Na_v_1.2 and Na_v_1.6 channels remain understudied. To overcome this challenge, we combined genome editing, super-resolution, and live-cell single-molecule imaging to probe subcellular composition, relative abundances, and trafficking dynamics of Na_v_1.2 and Na_v_1.6 in cultured mouse and rat neurons and in male and female mouse brain. We discovered a previously uncharacterized trafficking pathway that targets Na_v_1.2 to the distal axon of unmyelinated neurons. This pathway uses distinct signals residing in the intracellular loop 1 between transmembrane domain I and II to suppress the retention of Na_v_1.2 in the axon initial segment and facilitate its membrane loading at the distal axon. As mouse pyramidal neurons undergo myelination, Na_v_1.2 is gradually excluded from the distal axon as Na_v_1.6 becomes the dominant VGSC in the axon initial segment and nodes of Ranvier. In addition, we revealed exquisite developmental regulation of Na_v_1.2 and Na_v_1.6 localizations in the axon initial segment and dendrites, clarifying the molecular identity of sodium channels in these subcellular compartments. Together, these results unveiled compartment-specific localizations and trafficking mechanisms for VGSCs, which could be regulated separately to modulate membrane excitability in the brain.

**SIGNIFICANCE STATEMENT** Direct observation of endogenous voltage-gated sodium channels reveals a previously uncharacterized distal axon targeting mechanism and the molecular identity of sodium channels in distinct subcellular compartments.

## Introduction

Neurons are composed of several functionally distinct subcellular compartments, dendrites that receive electrochemical inputs, the cell body and axon initial segment (AIS) where action potentials initiate, and the distal axon harboring presynaptic components that send electrochemical output to downstream neurons. Voltage-gated sodium channels (VGSCs) are responsible for membrane depolarization and play a fundamental role in the generation and propagation of action potentials in excitable cells (Hodgkin and Huxley, 1952; Catterall et al., 2005). Four of nine paralogous VGSC genes (*SCN1A*, *SCN2A*, *SCN3A*, *SCN8A*) in the mammalian genome are enriched in the brain with distinct cell-type specificity and subcellular distributions (Vacher et al., 2008). Specifically, previous studies indicated the prominent presence of Na_v_1.2 and Na_v_1.6 in the AIS of excitatory neurons (Hu et al., 2009; Lorincz and Nusser, 2010). However, high-resolution imaging data that can simultaneously identify multiple VGSC paralogs are lacking to clarify their relative compositions in other subcellular compartments. For example, Na_v_1.6 was shown to localize in dendrites of hippocampal CA1 pyramidal neurons in adult rats via a highly sensitive electron microscopic immunogold technique, whereas Na_v_1.2 was not detected (Lorincz and Nusser, 2010). However, electrophysiology studies indicated that Na_v_1.2 plays a key role in Na_v_ currents at the somatodendritic region of cortical neurons in both young and adult mice (Spratt et al., 2019). Thus, direct observation of subcellular localization of Na_v_1.2 and Na_v_1.6 is necessary to clarify these discrepancies and advance our understanding of their physiological functions.

Currently, one of the major challenges to probe membrane proteins in the brain is lack of reliable labeling methods. Specifically, traditional immunolabeling is associated with the following limitations: (1) nonspecific cross-reaction, especially for antibodies against closely related channels and receptors, (2) insufficient sensitivity when the density of the target protein is low on the membrane, and (3) subcellular localization information is obscured by high packing density of neurites in the brain (Mikuni et al., 2016). Specifically, as widely expressed proteins such as VGSCs are intermingled within processes of many neurons, high resolution discrimination of their localization on the processes of any one neuron is difficult.

To address these limitations, here we combined CRISPR/Cas9 *in vivo* genome editing with high-affinity peptide tags V5 (GKPIPNPLLGLDST) or HA (YPYDVPDYA) and self-labeling tags (e.g., HaloTag) to label Na_v_1.2 and Na_v_1.6. Sparse cell labeling and high sensitivity of monoclonal antibodies enable us to reconstruct the subcellular localizations of channels with high spatial resolution in cortical excitatory neurons across different developmental stages. We found that Na_v_1.2 is highly enriched in the AIS, dendrites, and unmyelinated distal axon branches of pyramidal neurons during early development. As these neurons undergo myelination, Na_v_1.2 is excluded from the axon with an eventual establishment of Na_v_1.6 as the dominant VGSC at the AIS and nodes of Ranvier. Super resolution and live-cell single-molecule imaging in cultured neurons enabled real-time investigation of VGSC trafficking dynamics at nanometer scales. We found that once synthesized, Na_v_1.2 and Na_v_1.6 are sorted into distinct trafficking vesicles. Although localization of Na_v_1.2 and Na_v_1.6 to the AIS is dependent on Ankyrin G binding domain (ABD), as previously described (Garrido et al., 2003; Lemaillet et al., 2003), the targeting of Na_v_1.2 to unmyelinated fragments in the distal axon requires separate signals, previously not known, within the intracellular loop 1 (ICL1) between transmembrane domain I and II. Specifically, Na_v_1.2 ICL1 suppresses AIS retention and permits the membrane loading of Na_v_1.2 at the distal axon. Together, these results unveiled previously uncharacterized compartment-specific trafficking mechanisms for Na_v_1.2 and Na_v_1.6, which could be modulated independently to regulate channel-specific membrane composition and physiological functions in the brain.

## Materials and Methods

### Animals

Homozygous H11^LSL-Cas9^ CRISPR/Cas9 knock-in male mice (stock #027632, The Jackson Laboratory; Chiou et al., 2015) were crossed with wild-type C57Bl/6N females to get timed-pregnant heterozygous litters for *in utero* electroporation. Both male and female pups were used. All procedures were in accordance with protocols approved by the Janelia Research Campus Institutional Animal Care and Use Committee. Mice were housed in a 12 h light/dark cycle.

### DNA constructs

Knock-in constructs containing SpCas9, gRNA, and donor DNA were modified from PX551 and PX552 backbones, which were gifts from Feng Zhang (plasmid #60957 and #60958, Addgene; RRID:Addgene_60957, RRID:Addgene_60958, respectively). An EF1 promoter-driven spaghetti monster fluorescent protein with Flag tag (smFP_Flag; Viswanathan et al., 2015) cassette was inserted into PX552 construct to indicate successful plasmid transfection. All gRNAs were designed by the Web tool CHOPCHOP (Labun et al., 2019). The gRNA targeting sequence of rat *SCN2A* (site 1, extracellular loop between transmembrane segment 5 and 6 in domain I) is the following: 5′-TGGTACTGCCTTCAATAGGA-3′. The gRNA targeting sequence of mouse *SCN2A* (site 2, C terminus) is the following: 5′-GGACAAGGGGAAAGATATCA-3′. The gRNA targeting sequence of mouse *SCN8A* (C terminus, site 2) is the following: 5′-CCGACAAGGAGAAGCAGCAG-3′. Site 1 targeting was used for nonpermeabilized immunofluorescence staining of Na_v_1.2 in cultured hippocampal neurons. All other experiments were performed with site 2 targeting. Plasmids encoding mouse Na_v_1.2 (NP_001092768.1) and Na_v_1.6 (NP_001070967.1) were cloned by Gibson Assembly (NEB) with synthetic gBlocks gene fragments (Integrated DNA Technologies). Plasmids used for electrophysiological recording tests were designed based on the final sequences after SpCas9-mediated genetic knock-in.

### Primary culture of hippocampal neurons

We prepared dissociated hippocampal neurons from postnatal day (P)0 to P1 Sprague Dawley rat or C57Bl/6 mouse pups. Briefly, the hippocampi were dissected out and digested with papain (Worthington Biochemical). After digestion, the tissues were gently triturated and filtered with the cell strainer. The cell density was counted and ∼2.5 × 10^5^ cells were transfected with indicated constructs by using P3 Primary Cell 4D-Nucleofector X Kit (Lonza). After transfection, neurons were plated onto poly-D-lysine (PDL)-coated coverslips (Sigma-Aldrich) and maintained in NbActiv4 medium (BrainBits) at 37°C for indicated days.

### Immunofluorescence staining of cultured hippocampal neurons

Cultured neurons were fixed with 4% paraformaldehyde, permeabilized, and blocked with 10% fetal bovine serum, 1% Triton X-100 in PBS, incubated with primary antibodies against V5 tag (1:1000; catalog #R960-25, Thermo Fisher Scientific; RRID:AB_2556564; 1:1000; catalog #13202, Cell Signaling Technology; RRID:AB_2687461, respectively), HA tag (1:1000; catalog #3724, Cell Signaling Technology; RRID:AB_1549585), AnkG (1:1000; catalog #75-146, Antibodies Incorporated, NeuroMab clone N106/36; RRID:AB_10673030), MAP2 (1:5000; catalog #AB5622, Millipore; RRID:AB_91939), GFP (1:1000, catalog #A-11122, Thermo Fisher Scientific; RRID: AB_221569), or Flag tag (1:1000; catalog #ab1257, Abcam; RRID:AB_299216) overnight at 4°C. After washing with 10% fetal bovine serum in PBS, neuron samples were stained with Alexa Fluor conjugated secondary antibodies (1:1000, Thermo Fisher Scientific) and imaged with Nikon A1R confocal microscope or Zeiss LSM 880 Airyscan microscope. For actin staining, samples were stained with Alexa Fluor 594 phalloidin (1:1000; catalog #A12381, Thermo Fisher Scientific) and imaged with Leica SP8 STED microscope.

### *In utero* electroporation and histology

*In utero* electroporation was performed as previously described (Petreanu et al., 2009; Mikuni et al., 2016). In brief, timed-pregnant mouse (E13 for hippocampus and E15 for cerebral cortex) was anesthetized with 2 ∼ 2.5% isoflurane with an O_2_ flow rate of 0.5 ∼ 0.8 L/min. Before the surgery, a cotton-tip applicator was used to coat both eyes with puralube, and buprenorphine (0.1 mg/kg, i.p.; Bedford Laboratories) was administered for analgesia. DNA solution (1 ∼ 2 µl with a concentration of 1 µg/µl) was injected into the lateral ventricle via picospritzer. Electrical pulses (E13, 40 V for 50 ms, 8 times with 1 s intervals; E15, 45 V for 50 ms, 8 times with 1 s intervals) were delivered through ECM 830 electroporator. Ketaprofen (5 mg/kg, i.p.; Bedford Laboratories) was administered to reduce inflammation when the surgery was done and once a day for 2 d after the surgery.

After mouse pups were born and reached indicated ages, they were deeply anesthetized and perfused with 4% paraformaldehyde in 0.1 m phosphate buffer, pH 7.4. The brain was dissected out and postfixed overnight. After rinsed with PBS, coronal vibratome sections (70 µm in thickness) were made (VT1200S, Leica). The sections were permeabilized and blocked with 10% fetal bovine serum, 1% Triton in PBS, incubated with primary antibodies against V5 tag (1:1000; catalog #13202, Cell Signaling Technology, RRID:AB_2687461) and AnkG (1:1000; catalog #75-146, Antibodies Incorporated, NeuroMab clone N106/36; RRID:AB_10673030), myelin basic protein (MBP; 1:1000; catalog #SMI-99, Millipore Sigma; RRID:AB_2140491), Caspr (1:1000; catalog #75-001, Antibodies Incorporated, NeuroMab clone K65/35; RRID:AB_2083496), CaMKIIα (1:400; catalog #MA1-048, Thermo Fisher Scientific; RRID:AB_325403) or GAD67 (1:1000; catalog #MAB5406, Millipore Sigma; RRID:AB_2278725) overnight at 4°C. After washing with 10% fetal bovine serum in PBS, neuron samples were stained with Alexa Fluor conjugated secondary antibodies (Thermo Fisher Scientific) and imaged with a Zeiss 880 Airyscan microscope.

MBP staining images were used to quantify the percentage of labeled Na_v_1.2 or Na_v_1.6 in unmyelinated, myelinating, and myelinated neurons. Unmyelinated neurons are the ones with Na_v_1.2 or Na_v_1.6 signals and without MBP signals along the whole axon. Partially myelinated neurons are the ones with fragmented Na_v_1.2 or Na_v_1.6 signals (>10 µm) interspaced with MBP signals along the axon. Myelinated neurons are the ones with Na_v_1.2 or Na_v_1.6 signals in mature nodes of Ranvier (<10 µm) interspaced with MBP signals along the axon. The intensity distribution profiles along the AIS region and the intensity levels in dendrite of Na_v_1.2 and Na_v_1.6 in mouse cortical neurons at different ages were analyzed with Fiji software. The mean background intensity was subtracted before all further analysis.

### Whole-cell recording

HEK293 cells were cultured in DMEM with 10% fetal bovine serum in a 37°C incubator with 5% CO_2_ and were grown in 60 mm culture dishes. Plasmids encoding wild-type (WT) or V5-labeled Na_v_1.2, or wild-type Na_v_1.6 or V5-labeled Na_v_1.6 (4 µg) were cotransfected with *Scn1b* (2 µg), *Scn2b* (2 µg), and *Egfp* (0.3 µg) using Lipofectamine 2000 (Thermo Fisher Scientific). Whole-cell voltage-gated sodium (Na^+^) currents were measured 48 h after transfection at room temperature under voltage patch-clamp configuration with an Axopatch 200B amplifier (Molecular Devices) and sampled at 10 kHz and filtered at 2 kHz. Na^+^ currents were elicited with a 50 ms depolarization step from −100 mV with 5 mV increment at a holding potential of −100 mV. Steady-state inactivation was tested by a two-pulse protocol with the first pulse of 500 ms from −100 mV to −10 mV at 5 mV increment followed by a second pulse fixed at −10 mV. Gating activation and steady-state inactivation curves were obtained using a Boltzmann function as reported previously (Wang et al., 2021). The pipette solution contained the following (in mm): 35 CsF, 50 CsCl, 55 l-aspartic acid, 10 NaCl, 5 EGTA, 1 MgCl_2_, 4 Mg-ATP, 0.4 Na-GTP, and 10 HEPES, pH 7.3, with CsOH. The external solution contained the following (in mm): 120 NaCl, 5.4 KCl, 1.8 CaCl_2_, 1 MgCl_2_, 10 HEPES, 10 glucose, 20 tetraethylammonium chloride, pH 7.4, with NaOH. The access resistance was 7.9 ± 0.9 MΩ (WT) versus 6.9 ± 0.4 MΩ (V5 labeled; *t* test, *p* = 0.34) with 60–80% compensation, and 6.7 ± 0.6 MΩ (WT) versus 6.0 ± 0.5 MΩ (V5 labeled; *t* test, *p* = 0.43) with 80–90% compensation for Na_v_1.2 and Na_v_1.6, respectively.

### Pulse-chase single-molecule imaging

Transfected hippocampal neurons were plated onto an ultra-clean cover glass precoated with PDL and cultured for indicated days [days *in vitro* (DIV) 9 ∼ 10]. The cells were first incubated with 100 mm Janelia Fluor 646 HaloTag Ligand (JF646-HTL) for 1.5 ∼ 2 h. After washout, the labeling medium was replaced with 10 mm Janelia Fluor 549 HaloTag Ligand (JF549-HTL) for chase labeling (20 min for overexpression experiments, 40 min for knock-in experiments). After final washout, the cover glass was transferred to a live-cell culturing metal holder with phenol red-free NbActiv4 medium and mounted onto a Nikon Eclipse TiE Motorized Inverted microscope equipped with a 100× oil-immersion objective (NA = 1.49), an automatic TIRF/HILO (total internal reflection fluorescence/highly inclined and laminated optical) illuminator, a perfect focusing system, a tri-cam splitter, three EMCCDs (electron multiplying charge-coupled devices; iXon Ultra 897, Andor) and Tokai Hit environmental control (humidity, 37°C, 5% CO_2_). Adenylyl-imidodiphosphate (AMPPNP; 1 mm; catalog #A2647, Sigma-Aldrich) was added during whole imaging period for indicated experiment. Before single-molecule imaging, one snapshot JF646 image was captured to indicate the general labeling profile. For tracking JF549-labeled single molecules, we used a 561 nm laser with the excitation power of ∼150 W/cm^2^ at an acquisition time of 100 ms.

### Single-molecule localization, tracking, and diffusion analysis

For single-molecule localization and tracking, the spot localization (*x*, *y*) was obtained through 2D Gaussian fitting based on multiple target tracing (MTT) algorithms (Sergé et al., 2008). The localization and tracking parameters are listed in Table 1. The radius of confinement (RC) for each trajectory is calculated as the distance between the center of mass (the average position of all localizations in the trajectory) to the farthest localization from the center of mass. The differential probability density function (PDF) curve is obtained by subtraction of RC PDF distributions between conditions as indicated in each figure.

**Table 1.** Localization and tracking parameters for the MTT program

Parameter	Value
Localization error	1E-06
Deflation loops	3
Blinking (frames)	1
Maximum number competitors	3
Maximum diffusion coefficient (µm^2^/s)	3

Related to Materials and Methods.

### Experimental design and statistical analysis

The numbers of the experiments are indicated in the figure or figure legends, and the samples sizes were based on previous experience. Statistical analyses were performed with Prism 8 software (GraphPad). Comparisons among multiple groups were performed with one-way or two-way ANOVA and a *post hoc* Bonferroni test. Differences were considered to reach statistical significance when *p* < 0.05.

### Data availability

The materials and data that support the findings of this study are available from the corresponding author on request.

## Results

### Differential subcellular localizations of Na_v_1.2 and Na_v_1.6 in cultured hippocampal neurons

**Figure 1.**
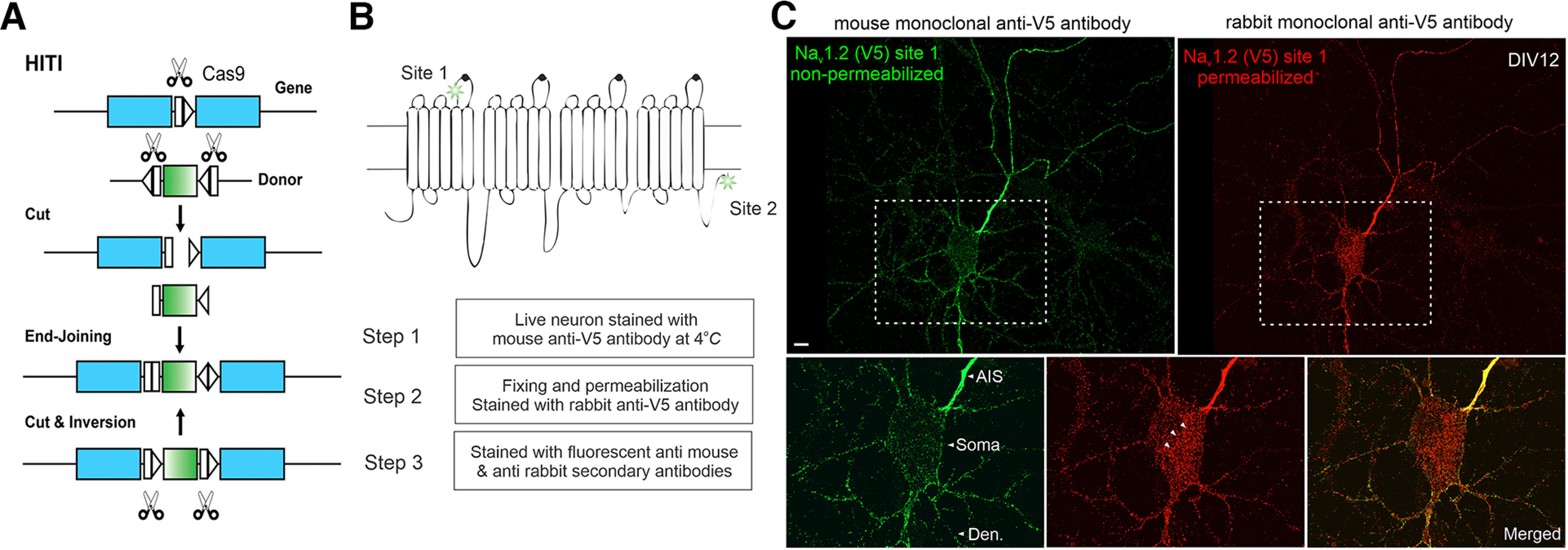
The HITI knock-in strategy and surface staining of V5-labeled Na_v_1.2 in cultured hippocampal neurons. ***A***, The schematics for the HITI strategy (Suzuki et al., 2016). The donor DNA fragment has two gRNA cutting sites flanking the tag cDNA. After cutting and end-joining, if the fragment is inserted into the genome in the right direction, the two gRNA cutting sites will be inactivated. If not, the cutting and end-joining process will continue until it is inserted in the right direction. ***B***, Top, Two gRNA targeting sites of Na_v_1.2 used in this study. Site 1 is at the extracellular loop between segment 5 and 6 of domain I. Site 2 is at the intracellular region near the C-terminus. Bottom, With V5 insertion at Site 1, we performed three-step immunofluorescence staining shown in ***C***. ***C***, Nonpermeabilized (green) and permeabilized immunofluorescence staining (red) images of Na_v_1.2 for the same neuron. Bottom, Zoomed-in views of the soma region (rectangle region with dashed lines). Arrowheads indicate labeled intracellular Na_v_1.2 vesicles (red), which were absent in the nonpermeabilized staining (green). Scale bar, 10 µm.

**Figure 3.**
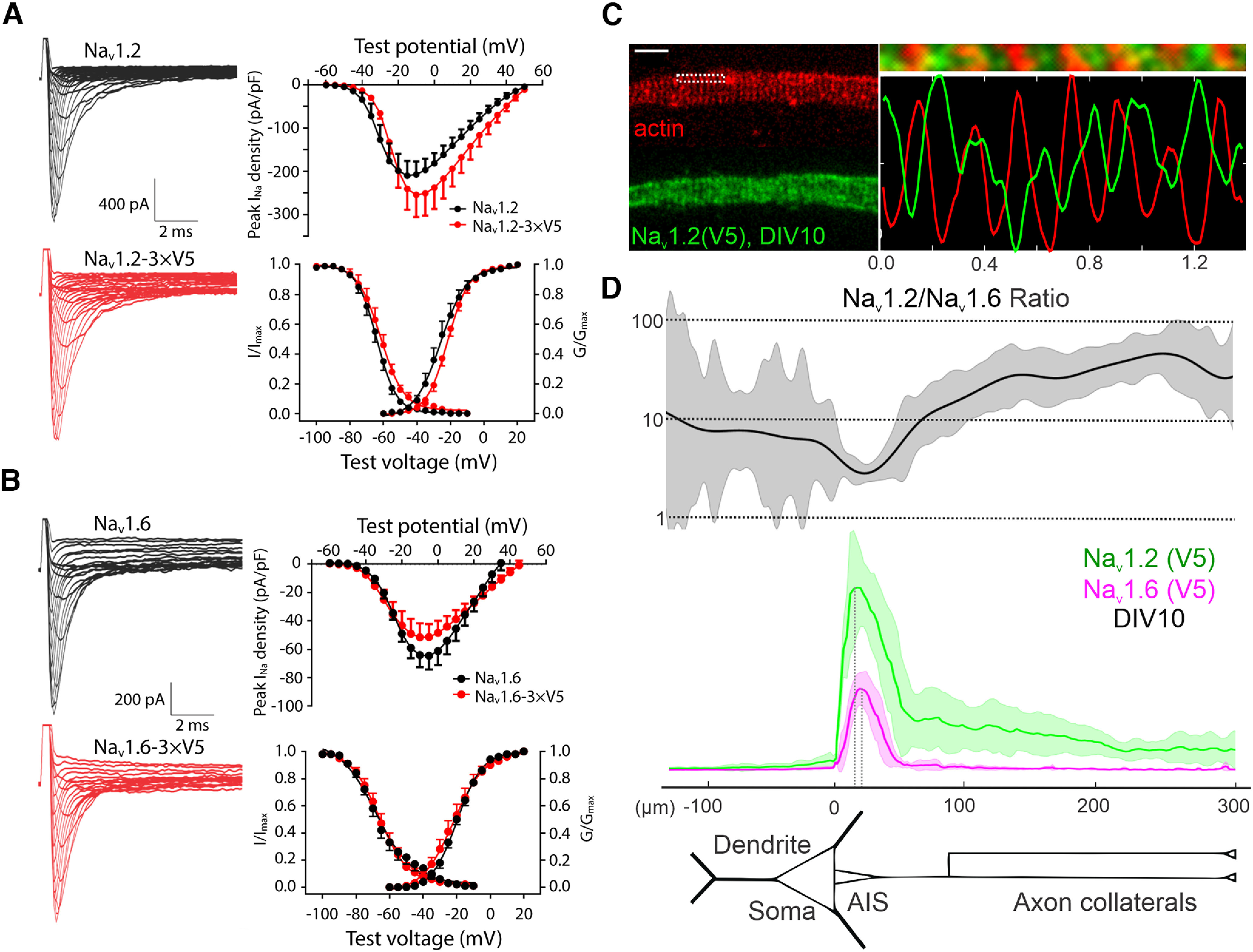
Electrophysiological properties and subcellular distribution profiles of V5-labeled Na_v_1.2 and Na_v_1.6. ***A***, WT (black) and V5-labeled (red) Na_v_1.2 in HEK293T cells. Left, Na_v_1.2 current examples. Right, Peak current density (top), channel activation (WT, *n* = 10; V5 labeled, *n* = 9), and steady-state inactivation (WT, *n* = 11; V5 labeled, *n* = 9) curves (bottom). ***B***, WT (black) and V5 labeled (red) Na_v_1.6 in HEK293T cells. Left, Na_v_1.6 current examples. Right, Peak current density (top), channel activation (WT, *n* = 11; V5 labeled, *n* = 12) and steady-state inactivation (WT, *n* = 13; V5 labeled, *n* = 11) curves (bottom). ***C***, Antiphase periodic striations of V5-labeled Na_v_1.2 and actin in the AIS region of cultured hippocampal neurons. Right, top, Zoomed-in view of the rectangle region with dashed lines in the left image. Bottom, The intensity curves of Na_v_1.2 (green) and actin (red) along the horizontal line. Scale bar, 1 µm. ***D***, Analysis of Na_v_1.2 (*n* = 12) and Na_v_1.6 (*n* = 14) relative intensities along the dendrite and axon of cultured hippocampal neurons. Top, Curve shows the Na_v_1.2/Na_v_1.6 intensity ratio calculated by using Na_v_1.2 and Na_v_1.6 intensity data (middle). Error bars (shadow areas) represent SD.

**Figure 2.**
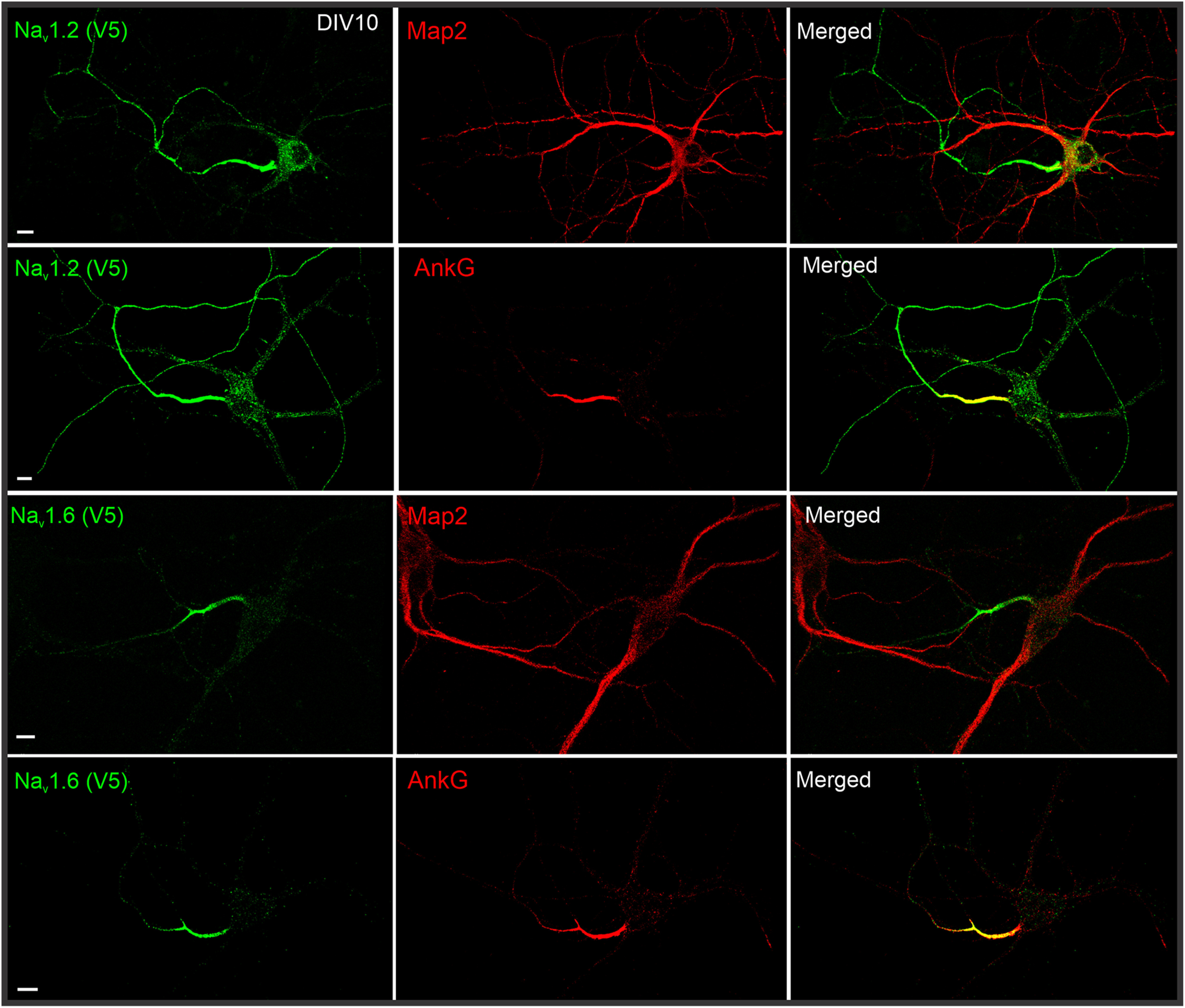
Immunostaining of V5-labeled Na_v_1.2 and Na_v_1.6 with MAP2 or AnkG in cultured hippocampal neurons. By cross-referencing with dendrite (MAP2) and AIS (AnkG) markers, we found that Na_v_1.2 is enriched in AIS, distal axon and dendrites, whereas Na_v_1.6 is mainly localized in the AIS region. Scale bar, 10 µm.

**Figure 4.**
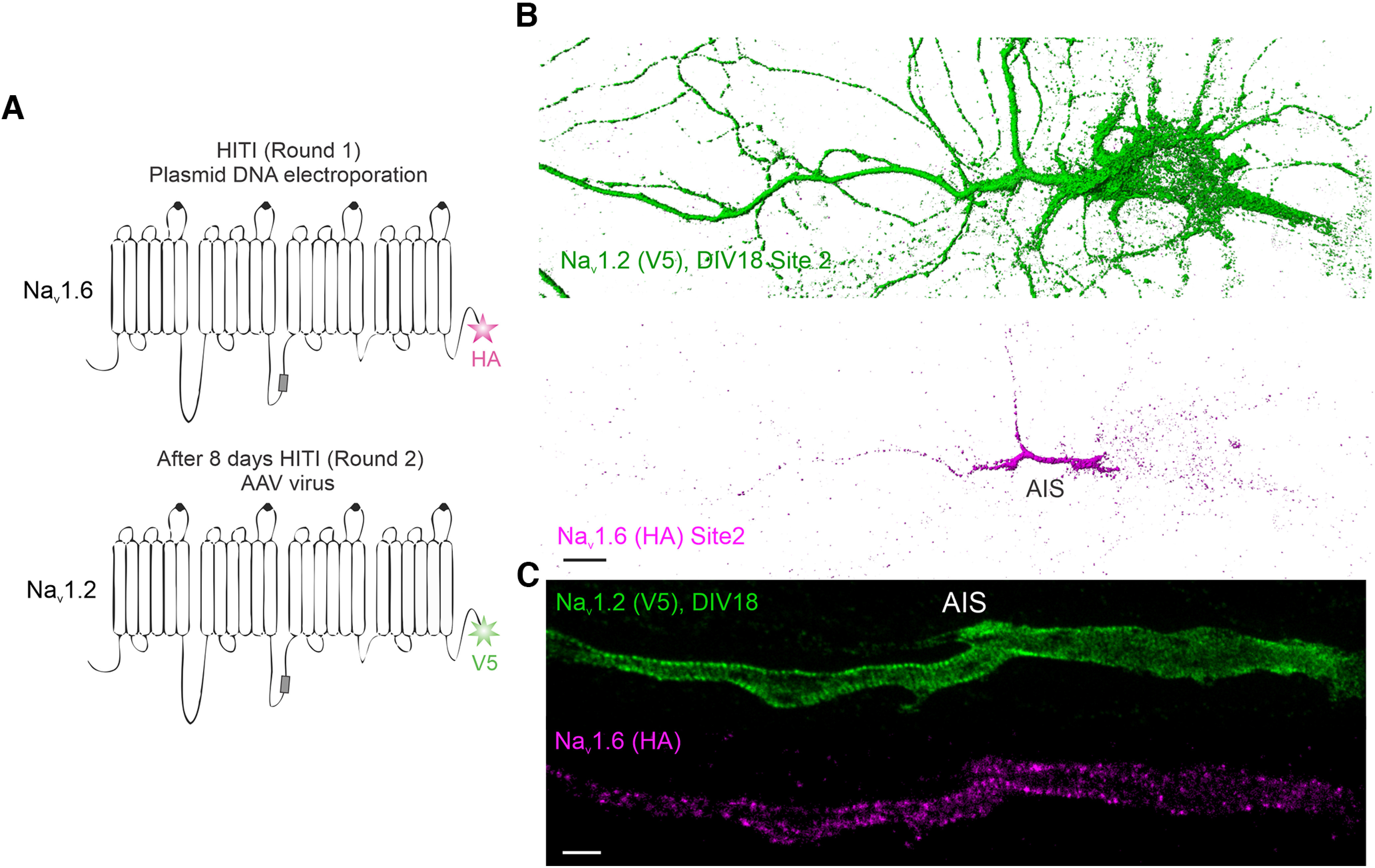
Dual labeling of Na_v_1.2 and Na_v_1.6 in the same neuron. ***A***, The double-labeling strategy of Na_v_1.2 and Na_v_1.6 in the same neuron. Through sequential plasmid DNA electroporation and AAV virus infection 8 d later, double labeling of Na_v_1.2 and Na_v_1.6 in the same neuron was achieved. ***B***, Labeling of Na_v_1.2 with V5 tag and Na_v_1.6 with HA tag in the same neuron (presentation, normal shading, Imaris). Scale bar, 10 µm. ***C***, Super-resolution STED imaging of labeled Na_v_1.2 and Na_v_1.6 in the AIS region. Scale bar, 1 µm. Movie 1 shows the 3D image of a representative double-knock-in neuron.

To probe the subcellular localizations of Na_v_1.2 and Na_v_1.6, we took advantage of a previously established homology-independent targeted integration (HITI) genome editing method (Suzuki et al., 2016; Fig. 1*A*) and tagged *Scn2a* (Na_v_1.2) and *Scn8a* (Na_v_1.6) with small peptide tags (V5 or HA). We reason that the small size of these tags would minimize the risk of perturbing their physiological functions. Indeed, V5 tag insertion at two independent locations of Na_v_1.2 (C terminus versus the extracellular loop between transmembrane segments 5 and 6 in domain I) gave rise to comparable subcellular localization patterns (Figs. 1*B*,*C*, 2). Single-cell recording confirmed that the tag insertion did not affect electrophysiological properties of Na_v_1.2 and Na_v_1.6 (Fig. 3*A*,*B*). Consistent with previous immunolabeling results (Xu et al., 2013), super-resolution stimulated emission depletion (STED) imaging revealed that tagged Na_v_1.2 and Na_v_1.6 formed ∼200 nm periodic striations that showed antiphased exclusion from actin rings at the AIS, further validating the labeling strategy (Figs. 3*C*, 4*C*).

To quantify relative abundances of Na_v_1.2 and Na_v_1.6 in distinct subcellular compartments, we tagged both channels with the V5 tag, followed by labeling and imaging under the same condition. This strategy allowed us to estimate their relative abundances in distinct neuronal compartments with high resolution. By cross-referencing with AIS (Ankyrin G) and dendrite (MAP2) markers, we found that both channels showed highest enrichment in the AIS (Fig. 2), consistent with previous reports (Hu et al., 2009; Lorincz and Nusser, 2010). Interestingly, however, we found that the relative abundance of Na_v_1.2 was much higher than Na_v_1.6 in the distal axon (Fig. 3*D*). To confirm that our observations were not influenced by cell-type-specific expression, we used sequential HITI genome editing and achieved dual labeling of Na_v_1.2 (V5) and Na_v_1.6 (HA) in the same cell population (Fig. 4*A*). It is important to note that although DIV10 neurons were processed for imaging under single HITI experiments, DIV18 neurons were used in the sequential HITI experiment as additional time was required for the second round of knock-in and downstream protein expression (Fig. 4*A*). Na_v_1.2 and Na_v_1.6 staining patterns in the dual labeling condition (Fig. 4*B*, Movie 1) were consistent with what was observed in separate populations with highest levels of Na_v_1.2 and Na_v_1.6 in the AIS and Na_v_1.2 as the dominant VGSC in the distal axon and dendrites (Fig. 3*D*). Additionally, because we performed HITI knock-in of Na_v_1.2 (V5) 1 week later than that of Na_v_1.6 (HA; Fig. 4*A*), this result also suggests that the timing of Na_v_1.2 expression does not notably affect its subcellular localization. Live-cell nonpermeable staining unambiguously confirmed that Na_v_1.2 was indeed inserted into cell membrane in the distal axon and the somatodendritic region of cultured hippocampal pyramidal neurons (Fig. 1*B*,*C*). These high-resolution imaging data reveal differential compartment-specific localization patterns of Na_v_1.2 and Na_v_1.6 in cultured neurons.

Movie 1. Related to Figure 4. A representative cultured hippocampal neuron with double labeling of Na_v_1.2 (V5, green) and Na_v_1.6 (HA, red) costained with Flag (blue). 10.1523/JNEUROSCI.0086-22.2022.video.1

### Characterization of Na_v_1.2 and Na_v_1.6 subcellular localizations *in vivo*

**Figure 5.**
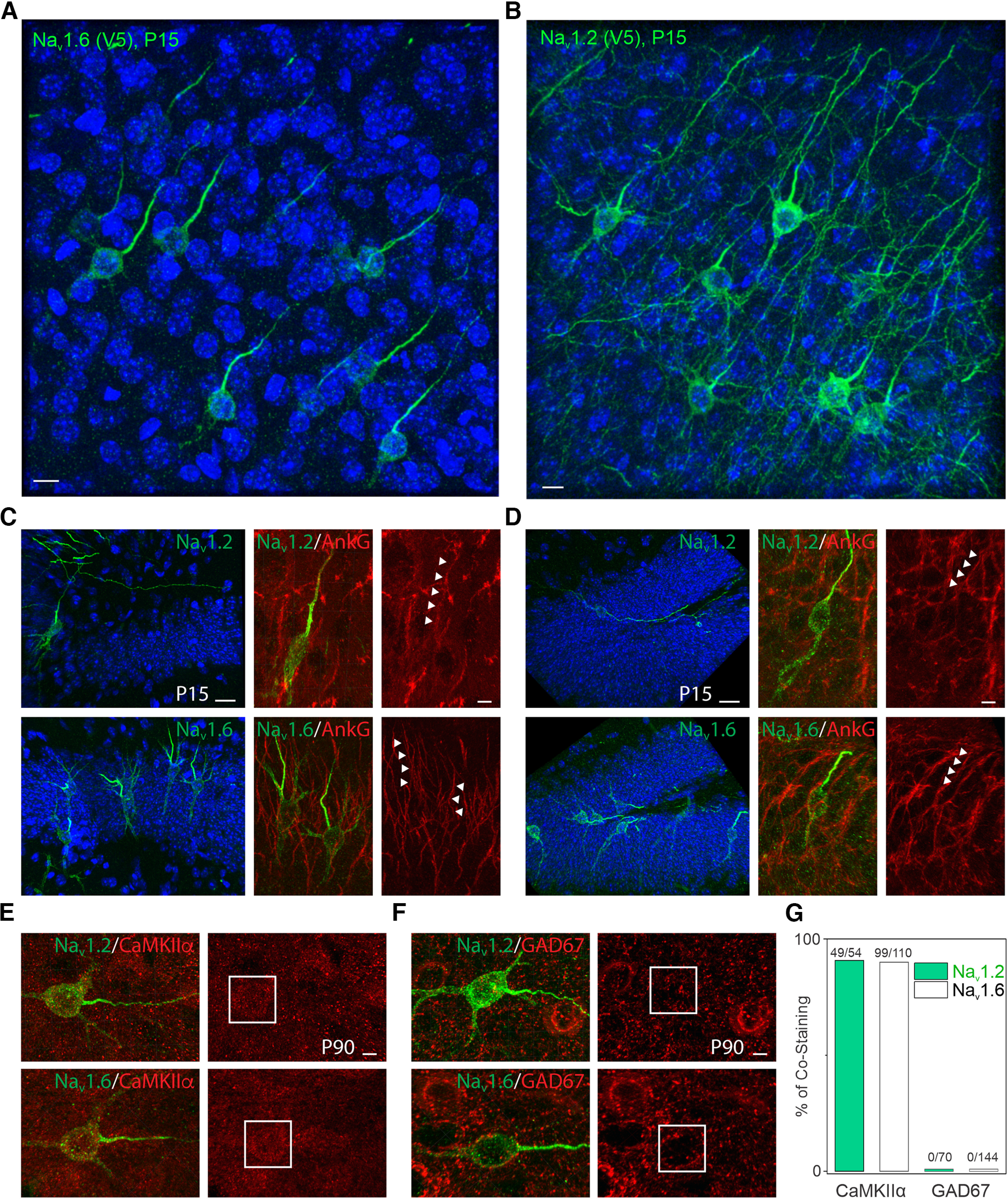
Differential subcellular distribution and cell-type-specific expression of Na_v_1.2 and Na_v_1.6 in the mouse brain. ***A***, ***B***, Representative images of Na_v_1.6 (***A***) and Na_v_1.2 (***B***) labeled with V5 tag in the cortex. Blue channel shows the Hoechst stain. Scale bar, 10 µm. ***C***, ***D***, Representative images of Na_v_1.2 and Na_v_1.6 labeled with V5 tag in CA1 (***C***) and dentate gyrus (***D***) of the hippocampus. Left, Blue channel shows the Hoechst stain. Scale bar, 20 µm. Right, Zoomed-in images costained with AnkG. Arrowheads indicate Na_v_1.2 or Na_v_1.6-positive region with AnkG signals. Scale bar, 5 µm. ***E***, ***F***, Double-immunofluorescence staining of V5-labeled Na_v_1.2 or Na_v_1.6 with CaMKIIα (***E***) or GAD67 (***F***) in the mouse cortex. Rectangles highlight the soma regions of Na_v_1.2 or Na_v_1.6-positive neurons. Scale bar, 5 µm. ***G***, The positive ratio of Na_v_1.2 or Na_v_1.6 knock-in cells in CaMKIIα-positive excitatory or GAD67-positive inhibitory neurons.

To map Na_v_1.2 and Na_v_1.6 localizations *in vivo*, we used *in utero* electroporation to deliver the HITI construct into heterozygous H11-SpCas9 mouse embryos expressing Cas9 in all cell types (Chiou et al., 2015). Because both Na_v_1.2 and Na_v_1.6 were tagged with the V5 peptide, we were able to estimate their relative abundances in individual processes of sparsely labeled neurons across large distances at different developmental stages (P15; P30, ∼1 month; P90, ∼3 months) in mouse neocortex (Fig. 5*A*,*B*). It is worth noting that in this assay Na_v_1.2 and Na_v_1.6 levels are obtained by averaging aggregated data from Na_v_1.2 and Na_v_1.6 positive knock-in neurons. In agreement with previous reports (Hu et al., 2009; Tian et al., 2014; Yamagata et al., 2017), we found that the majority of Na_v_1.2 or Na_v_1.6 positive knock-in neurons (>90%) were CaMKIIα-positive excitatory neurons (Fig. 5*E*,*G*), and we only analyzed imaging data from these neurons.

**Figure 6.**
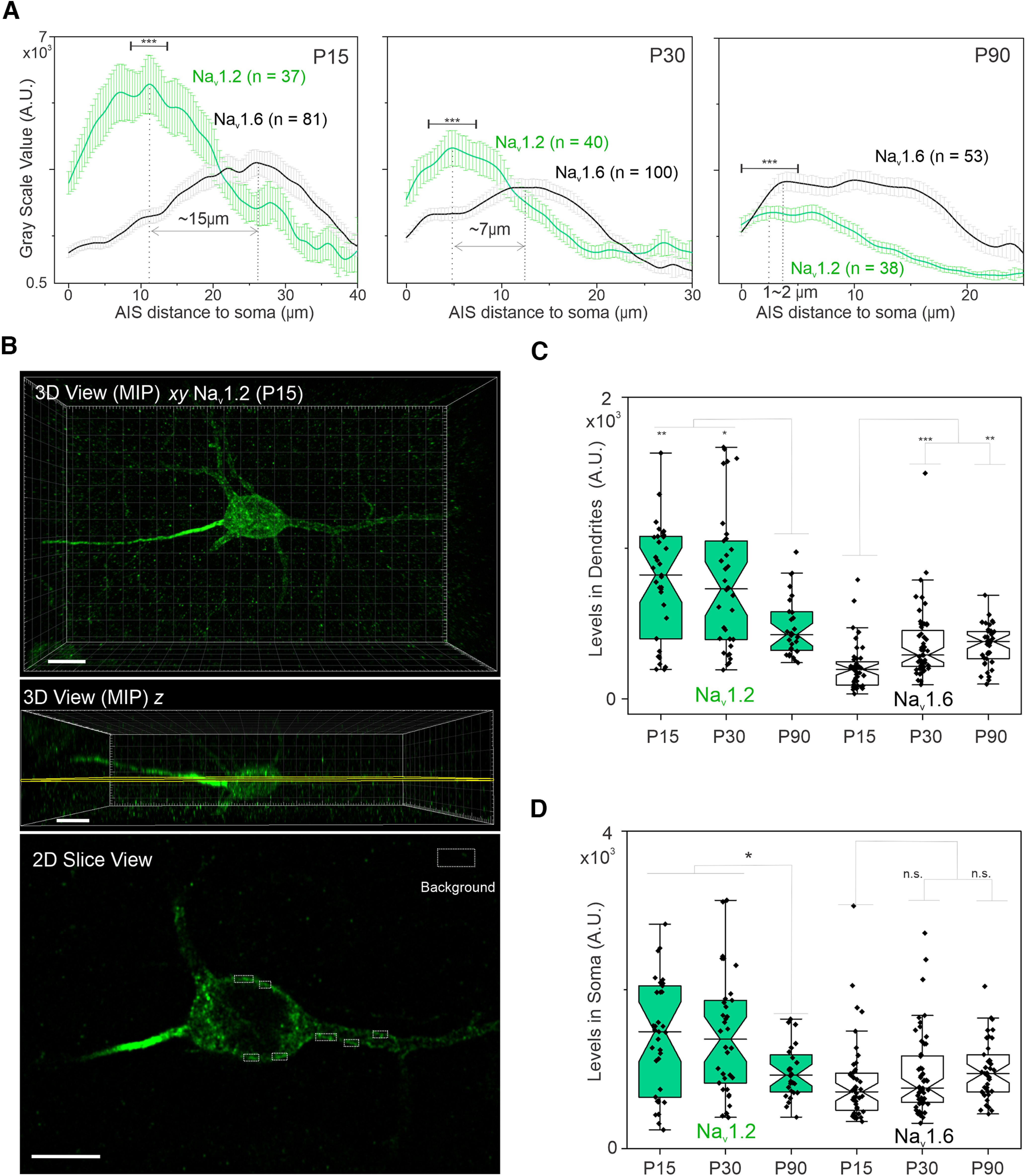
Quantification of Na_v_1.2 and Na_v_1.6 levels in the AIS, soma, and dendrites of neocortical neurons. ***A***, Intensity measurements of V5-labeled Na_v_1.2 and Na_v_1.6 in the AIS of neocortical neurons at P15, P30, and P90. Error bars represent SEM; *n* indicates the number of cells analyzed; *p* < 0.0001 for all the comparisons among the intensity peak regions (∼5 µm, indicated regions above the curves) of Na_v_1.2(P15), Na_v_1.2(P30), and Na_v_1.2(P90). ***B***, Top and middle, Three-dimensional views of a neocortical neuron with Na_v_1.2 V5 labeling at P15. MIP, Maximum intensity projection. To quantify Na_v_1.2 in the soma and dendrites, we used a 2D slice view (bottom) with the slicing (middle) and selectively measured the above-background fluorescence intensities (dotted boxes) along the cell edge. The background signals were calculated from a random blank area indicated by the larger dotted box. Scale bar, 10 µm. ***C***, Box plots of intensity measurements of V5-labeled Na_v_1.2 and Na_v_1.6 in dendritic regions of neocortical neurons at different ages. In the box chart, top and bottom error bars represent the 95th and 5th percentiles, respectively; box represents the range from 25th to 75th percentile; center line represents the median. Na_v_1.2(P15) versus Na_v_1.2(P90), *p* = 0.0032; Na_v_1.2(P30) versus Na_v_1.2(P90), *p* = 0.0207; Na_v_1.6(P15) versus Na_v_1.6(P30), *p* = 0.0003; Na_v_1.6(P15) versus Na_v_1.6(P90), *p* = 0.0011. ***D***, Box plots of intensity measurements of V5-labeled Na_v_1.2 and Na_v_1.6 in the soma of neocortical neurons at different ages. Na_v_1.2(P15) versus Na_v_1.2(P90), *p* = 0.0142; Na_v_1.2(P30) versus Na_v_1.2(P90), *p* = 0.0454; Na_v_1.6(P15) versus Na_v_1.6(P30), n.s.; Na_v_1.6(P15) versus Na_v_1.6(P90), n.s.

We found that both Na_v_1.2 and Na_v_1.6 were enriched at the AIS (Fig. 5*A–D*) with much higher levels of Na_v_1.2 in the distal axon and dendrites during early developmental stage (P15; Fig. 6*A*,*C*), similar to our observations in cultured hippocampal neurons. We also noticed that Na_v_1.2 was enriched at the proximal part of the AIS, whereas Na_v_1.6 was concentrated at the distal part of the AIS with an ∼15 µm gap between their concentration peaks at P15 (Fig. 6*A*), consistent with a previous report (Hu et al., 2009). Interestingly however, Na_v_1.2 levels decreased significantly at the proximal AIS with the Na_v_1.6 concentration peak shifting closer to the soma at later developmental stages (P30 and P90; Fig. 6*A*).

We were also able to confirm the localization of Na_v_1.2 in dendrites of neocortical pyramidal neurons *in vivo* (Fig. 5*B*) as high-resolution Airyscan imaging and optical sectioning enabled visualization and estimation of Na_v_1.2 and Na_v_1.6 levels along the cell edge (presumably the cell membrane) of the soma and dendrites (Fig. 6*B*). We found that Na_v_1.2 showed much higher enrichments than Na_v_1.6 in the dendrites during early development, and its concentration gradually decreased, accompanied by a modest increase of Na_v_1.6 levels as mice matured (Fig. 6*C*,*D*).

### Myelination status as a key indicator of Na_v_1.2 and Na_v_1.6 localization patterns

**Figure 7.**
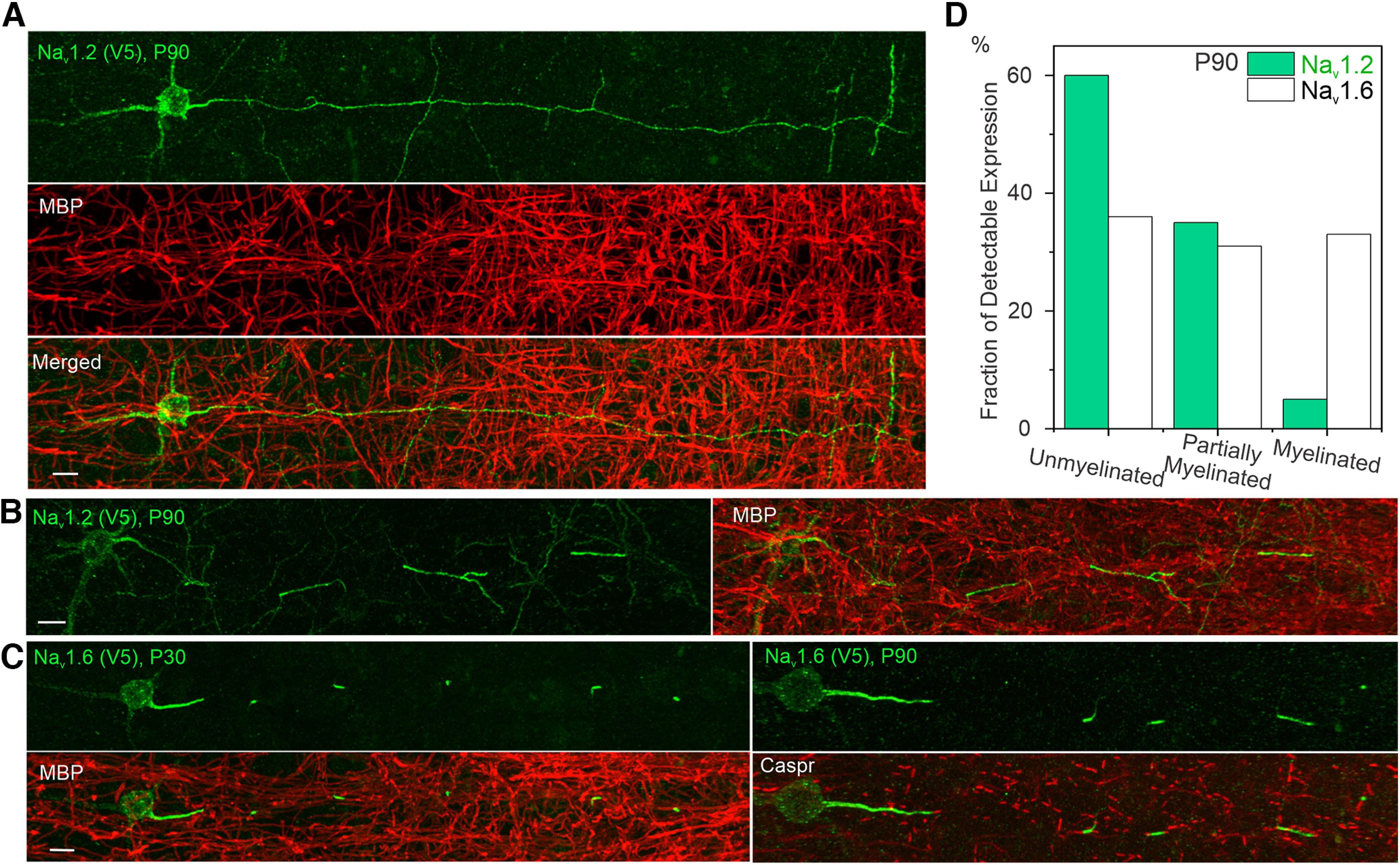
The relationship between Na_v_1.2 and Na_v_1.6 localizations and the myelination status of pyramidal neurons. ***A***, ***B***, Double immunostaining of V5-labeled Na_v_1.2 with MBP (red) in unmyelinated neuron (***A***) and partially myelinated neuron (***B***). Scale bar, 10 µm. ***C***, Double immunostaining of V5-labeled Na_v_1.6 with MBP (left) and Caspr (right). Scale bar, 10 µm. ***D***, The percentage of unmyelinated, partially myelinated, and myelinated neurons in Na_v_1.2- or Na_v_1.6-positive knock-in neurons. The number of cells analyzed, Na_v_1.2, 60; Na_v_1.6, 97. Movies 2, 3, 4 show 3D image details.

One consistent observation across all developmental stages was that the axonal coverage by Na_v_1.2 was largely uninterrupted in neurons with high Na_v_1.2 expression levels, suggesting that these neurons are unmyelinated (Fig. 7*A*, Movie 2). Indeed, when using MBP to costain samples, we found that Na_v_1.2 was preferentially expressed (∼60%) in unmyelinated neurons, with a smaller fraction (∼35%) of detectable expression in partially myelinated neurons, and the lowest fraction (∼5%) in fully myelinated neurons (Fig. 7*A*,*B*,*D*). In contrast, Na_v_1.6 had similar fractions of detectable expression across all three populations (Fig. 7*D*). It is important to note here that the fraction is defined as the percentage of unmyelinated, partially myelinated, and myelinated neurons in Na_v_1.2- or Na_v_1.6-positive knock-in neurons. In addition, we found that the axonal coverage by Na_v_1.6 was restricted to the AIS and nodes of Ranvier (Fig. 7*C*, Movies 3, 4), whereas Na_v_1.2 broadly covered the AIS and unmyelinated axonal fragments in the distal axon (Fig. 7*A*, Movie 2). Our results strongly suggest that localizations and expression levels of these two channels alter with the myelination status of the pyramidal neuron, which is consistent with previous studies (Boiko et al., 2001). Specifically, upon myelination, Na_v_1.2 is gradually excluded from the distal axon with an eventual establishment of Na_v_1.6 as the dominant VGSC at the AIS and nodes of Ranvier in fully myelinated neurons.

Movie 2. Related to Figure 7. 3D Airyscan image of Na_v_1.2 knock-in neurons (V5, green) in mouse cortex costained with MBP (red) shows its continuous distribution along distal axons without myelin coverage. 10.1523/JNEUROSCI.0086-22.2022.video.2

Movie 3. Related to Figure 7. Three-dimensional Airyscan image of Na_v_1.6 knock-in neurons (V5, green) in mouse cortex costained with MBP (red) shows its presence in myelinated neurons. 10.1523/JNEUROSCI.0086-22.2022.video.3

Movie 4. Related to Figure 7. Three-dimensional Airyscan image of Na_v_1.6 knock-in neurons (V5, green) in mouse cortex costained with Caspr (red) shows its localization at nodes of Ranvier. 10.1523/JNEUROSCI.0086-22.2022.video.4

### Compartment-specific targeting mechanisms for Na_v_1.2 and Na_v_1.6

**Figure 8.**
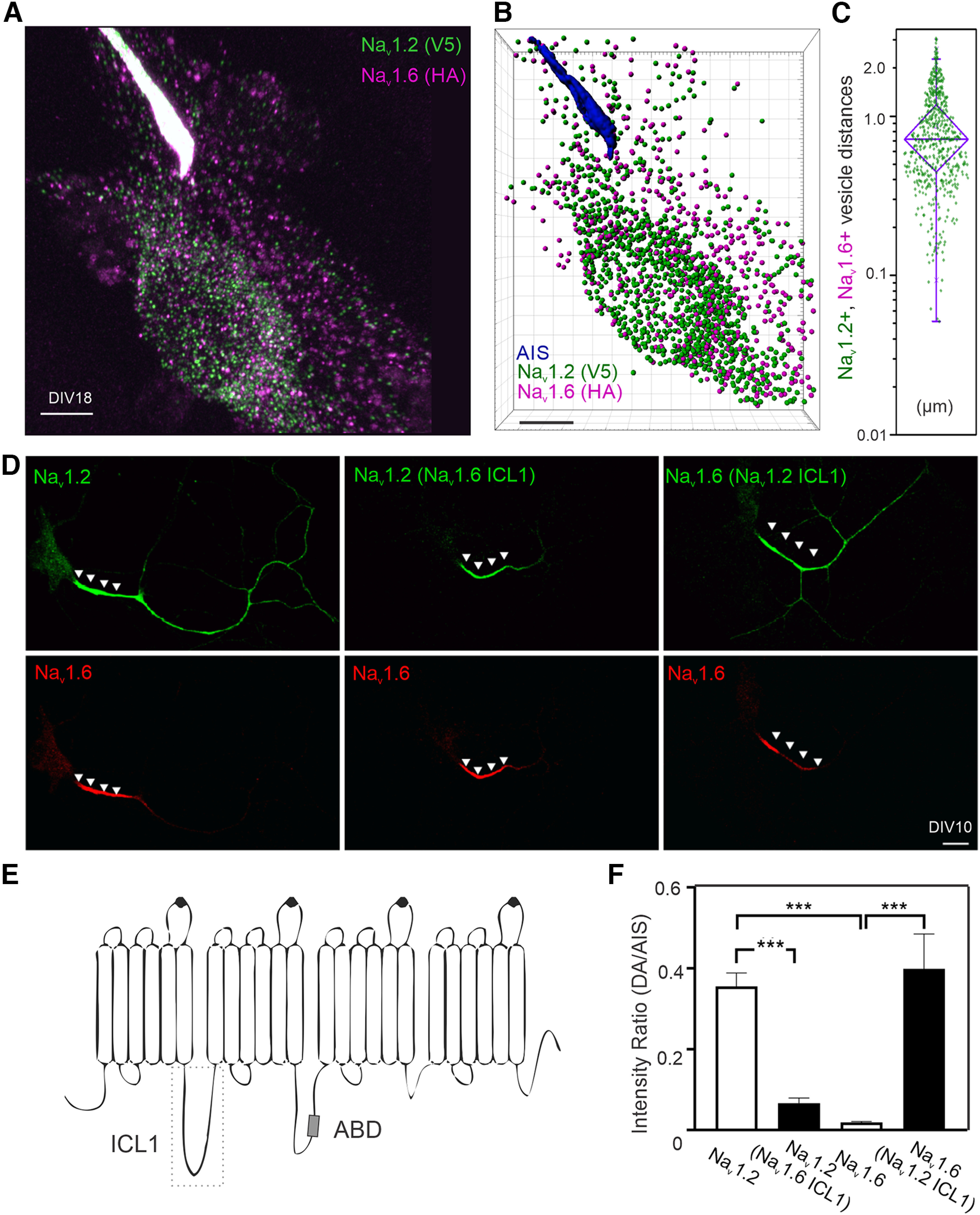
Compartment-specific targeting mechanisms for Na_v_1.2 and Na_v_1.6. ***A***, Airyscan imaging of Na_v_1.2- and Na_v_1.6-positive vesicles in the same neuron. Movie 5 shows 3D image details. ***B***, Computer-aided segmentation of Na_v_1.2 and Na_v_1.6 vesicles. Scale bar, 5 µm. ***C***, The distribution of physical distances between Na_v_1.2- and Na_v_1.6-positive vesicle populations. In the box chart, top and bottom error bars represent the 95th and 5th percentiles, respectively; triangle represents the range from the 25th to 75th percentile; center line represents the median. ***D***, The distribution patterns of exogenously expressed Na_v_1.2 and Na_v_1.6 in culture hippocampal neurons. Left, Localization patterns of wild-type Na_v_1.2 (green, V5) and Na_v_1.6 (red, HA). Middle, Na_v_1.2 with Na_v_1.6 ICL1 showed minimal enrichment at the distal axon. Right, Na_v_1.6 with Na_v_1.2 ICL1 gained access to the distal axon. Arrowheads indicate the AIS region. Scale bar, 20 µm. ***E***, Rectangle region with dashed lines shows the ICL1 region of Na_v_1.2 and Na_v_1.6. ABD is indicated by a small gray rectangle. ***F***, Na_v_1.2 with Na_v_1.6 ICL1 showed dramatic less enrichment in the distal axon. Conversely, Na_v_1.6 with Na_v_1.2 ICL1 gains the ability to localize to the distal axon. Error bars represent SEM. Na_v_1.2, *n* = 10 versus Na_v_1.2(Na_v_1.6 ICL1), *n* = 8, *p* = 0.0005; Na_v_1.2 versus Na_v_1.6, *p* < 0.0001; Na_v_1.6, *n* = 10 versus Na_v_1.6(Na_v_1.2 ICL1), *n* = 13, *p* < 0.0001.

The differential subcellular localization patterns of Na_v_1.2 and Na_v_1.6 that we observe *in vitro* and *in vivo* prompted us to image vesicle populations associated with their trafficking. Unexpectedly, super-resolution Airyscan imaging and computer-aided segmentation revealed negligible colabeled fraction between Na_v_1.2 and Na_v_1.6 positive trafficking vesicles (Fig. 8*A–C*, Movie 5), suggesting that once synthesized, Na_v_1.2 and Na_v_1.6 are sorted into distinct vesicle pools potentially coupled with separate trafficking and membrane loading pathways.

Movie 5. Related to Figure 8. Raw Airyscan image of V5-labeled Na_v_1.2 (green) and HA-labeled Na_v_1.6 (purple) vesicles in the soma region of a cultured hippocampal neuron and their distributions after segmentation. Scale bar, 5 µm. 10.1523/JNEUROSCI.0086-22.2022.video.5

**Figure 9.**
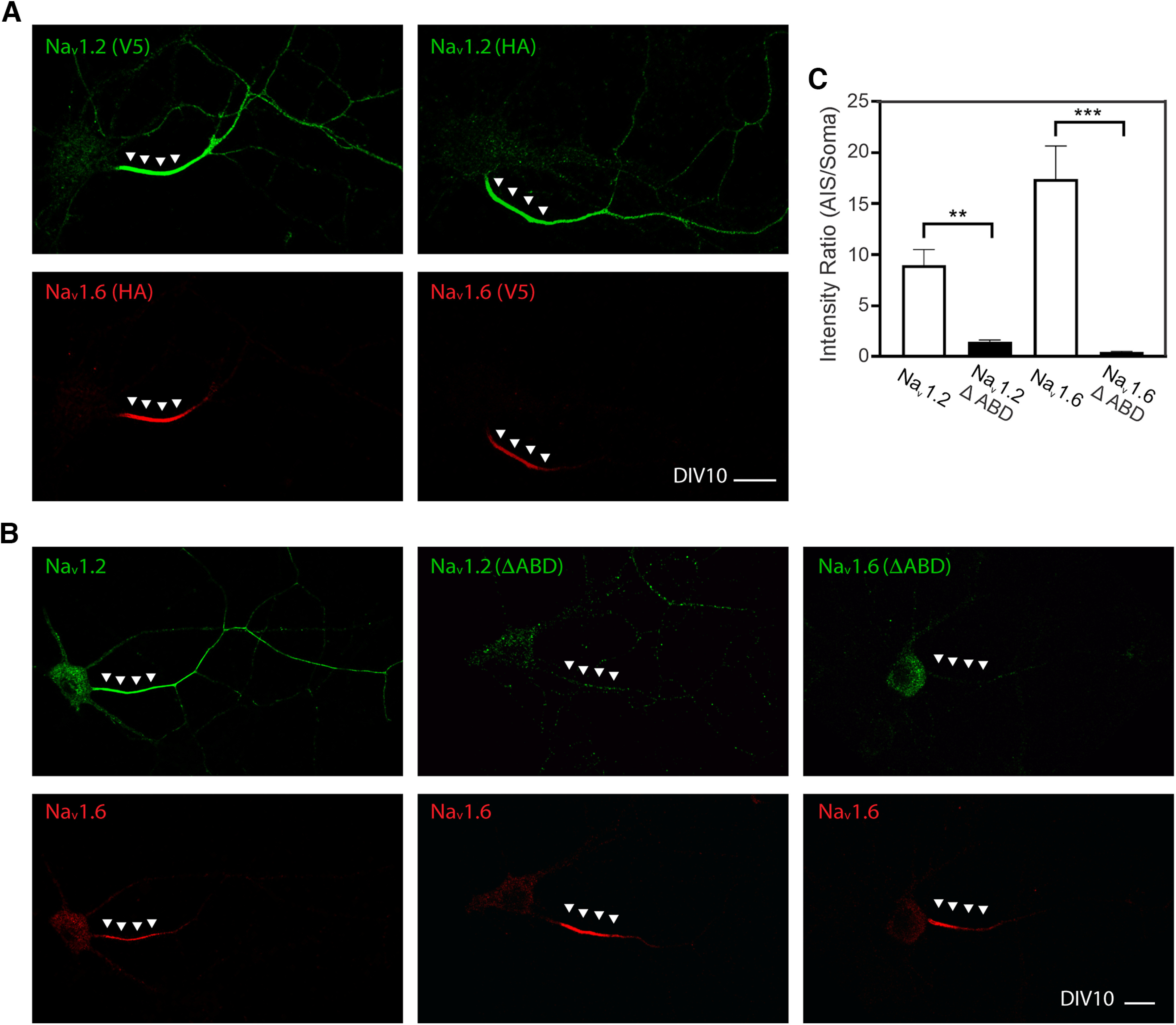
ABD deletion greatly reduced Na_v_1.2 and Na_v_1.6 levels in the AIS. ***A***, Switching V5 and HA tags to label Na_v_1.2 and Na_v_1.6 didn't affect their subcellular distribution patterns. ***B***, After ABD deletion, Na_v_1.2 and Na_v_1.6 localizations in the AIS are dramatically decreased. Green, V5; red, HA. Arrowheads in ***A*** and ***B*** indicate the AIS region. Scale bars, ***A***, ***B***, 20 µm. ***C***, Deletion of ABD abolished enrichment of Na_v_1.2 and Na_v_1.6 in AIS. Error bars represent SEM. Na_v_1.2, *n* = 10 versus Na_v_1.2(ΔABD), *n* = 13, *p* = 0.0049; Na_v_1.6, *n* = 10 versus Na_v_1.6(ΔABD), *n* = 17, *p* < 0.0001.

**Figure 10.**
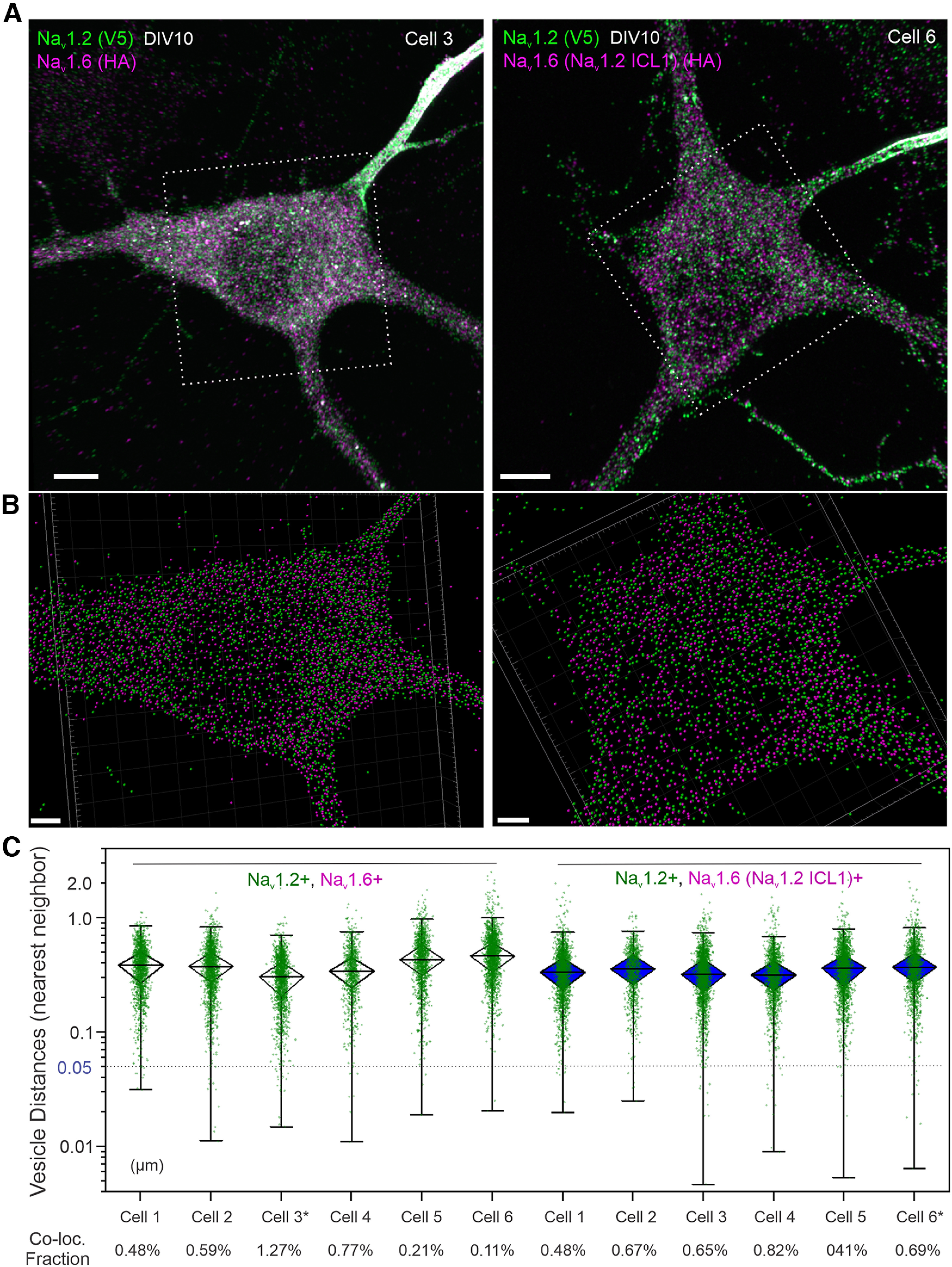
Characterizing the role of ICL1 in protein sorting. ***A***, Left, Airyscan images of DIV10 neurons double labeled with Na_v_1.2 (V5, green) and Na_v_1.6 (HA, magenta); or, right, Na_v_1.2 (V5, green) and Na_v_1.6(Na_v_1.2 ICL1) (HA, magenta) by cotransfection with corresponding plasmids. Scale bar, 5 µm. ***B***, Computer-aided vesicle segmentation. The frame corresponds to the dotted box in ***A***. Scale bar, 2 µm. ***C***, The distribution of physical distances (nearest neighbor) between Na_v_1.2- and Na_v_1.6-positive vesicle populations (Na_v_1.2 as the origin). In the box chart, top and bottom error bars represent the 95th and 5th percentiles, respectively; triangle represents the range from the 25th to 75th percentile; center line represents the median. The 50 nm dotted line is used as the cutoff for colocalization (Co-loc.). Bottom, The colocalization fraction (percentage) is indicated for each cell.

**Figure 11.**
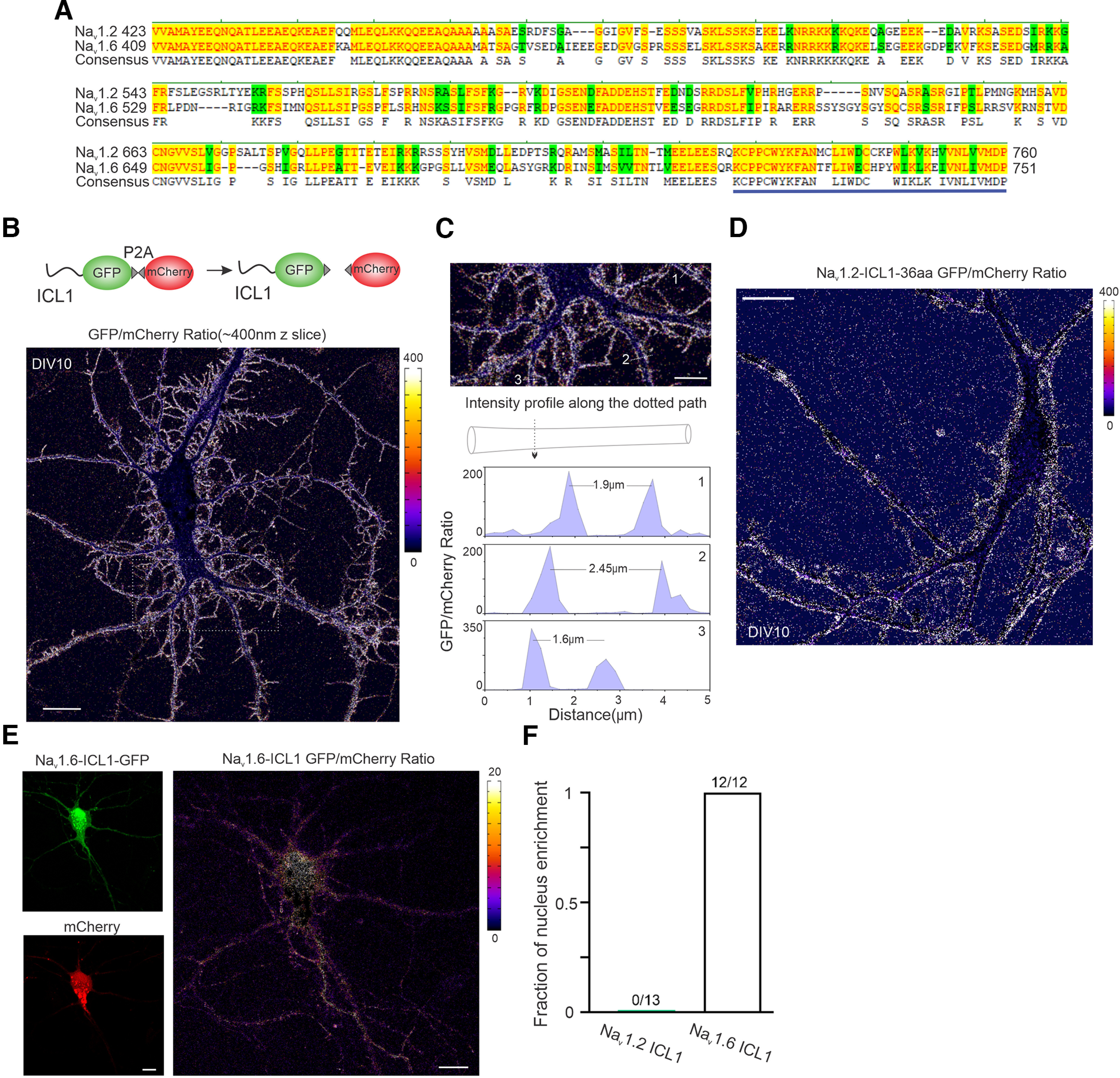
Na_v_1.2 ICL1 and Na_v_1.6 ICL1 target GFP to membrane and the nucleus, respectively. ***A***, Protein sequence alignment of Na_v_1.2 and Na_v_1.6 ICL1 region. Bold blue line highlights identified 36aa region. ***B***, Top, Illustration of the ratiometric localization analysis. After translation, ICL1-GFP and mCherry proteins are separated because of ribosome skipping at P2A. A representative intensity ratio (Na_v_1.2 ICL1-GFP/mCherry) image showed the localization of Na_v_1.2 ICL1 to the membrane sites. Right, Color bar indicates the ratio level. Scale bar, 20 µm. ***C***, Top, The enlarged view of the rectangle region with dashed lines in ***B***. Three neurites were chosen to analyze their intensity profiles along the dotted paths; bottom, the results are shown. Scale bar, 10 µm. ***D***, A representative intensity ratio image of Na_v_1.2 ICL1-36aa GFP/mCherry showed the specific distribution of Na_v_1.2 ICL1-36aa along the membrane. Right, Color bar indicates the ratio level. Scale bar, 20 µm. ***E***, Representative raw (left) and intensity ratio (right) images of Na_v_1.6 ICL1-GFP/mCherry suggest a nucleus enrichment of Na_v_1.6-ICL1-GFP. Right, Color bar indicates the ratio level. Scale bar, 20 µm. ***F***, The percentage of neurons showed nucleus localization of GFP signals.

To study the underlying mechanism, we established a two-color imaging assay in which Na_v_1.2 and Na_v_1.6 were coexpressed in cultured hippocampal neurons, where their localization patterns can be directly compared in distinct subcellular compartments. We found that exogenously expressed Na_v_1.2 (V5) and Na_v_1.6 (HA) displayed similar localization patterns as endogenous knock-in proteins, with both VGSCs enriched in AIS and higher levels of Na_v_1.2 in distal axon (Fig. 8*D*). Swapping labeling tags (Na_v_1.2 (HA) and Na_v_1.6 (V5)) did not affect these localization patterns (Fig. 9*A*), confirming that anti-HA and anti-V5 monoclonal antibodies have similar levels of detection sensitivity. Because of conserved sequence homology in membrane embedded domains, we focused on intracellular loops, which have greater sequence divergences. By extensive domain swapping between Na_v_1.2 and Na_v_1.6, we identified the ICL1 between transmembrane domains I and II (Fig. 8*E*, see Fig. 11*A*) as the key determinant for selective enrichment of Na_v_1.2 in the distal axon. Specifically, Na_v_1.2 with Na_v_1.6 ICL1 displayed the same localization pattern as Na_v_1.6, with very low enrichment in the distal axon (Fig. 8*D*,*F*). Conversely, Na_v_1.6 harboring ICL1 from Na_v_1.2 showed selective enrichment in the distal axon as Na_v_1.2, even after normalization of the expression level in the distal axon to protein levels in the AIS (Fig. 8*D*,*F*). This result suggests that ICL1 selectively affects the protein localization rather than the protein expression level. Importantly, we found that ICL1 is not responsible for differential sorting of these two VGSCs as Na_v_1.6 with Na_v_1.2 ICL1 still displayed minimal colocalization with Na_v_1.2 in the soma (Fig. 10). Consistent with previous reports (Garrido et al., 2003; Lemaillet et al., 2003; Gasser et al., 2012), we found that ABD deletion led to the loss of enrichment of Na_v_1.2 or Na_v_1.6 in the AIS (Fig. 9*B*,*C*). These results suggest that Na_v_1.2 ICL1 contains previously uncharacterized distal axon targeting and membrane loading signals.

To further dissect the function of ICL1, we fused it to GFP-P2A-mCherry. Interestingly, Na_v_1.2 ICL1 itself was able to broadly target GFP to cell membrane across different compartments (soma, axon, and dendrites; Fig. 11*B*,*C*), whereas, surprisingly, Na_v_1.6 ICL1-GFP signals were largely in the nucleus (Fig. 11*E*,*F*), consistent with previous reporting of a nuclear localization signal within this region (Onwuli et al., 2017). Using this assay, we further determined that a 36 amino acid region (AA725-760) within Na_v_1.2 ICL1 was sufficient for anchoring GFP to membrane (Fig. 11*D*). Together, these results suggest that Na_v_1.2 is targeted to the AIS and the distal axon by distinct trafficking mechanisms with one mediated by ABD and the other by ICL1.

### A model for targeting Na_v_1.2 to unmyelinated fragments in the distal axon

**Figure 12.**
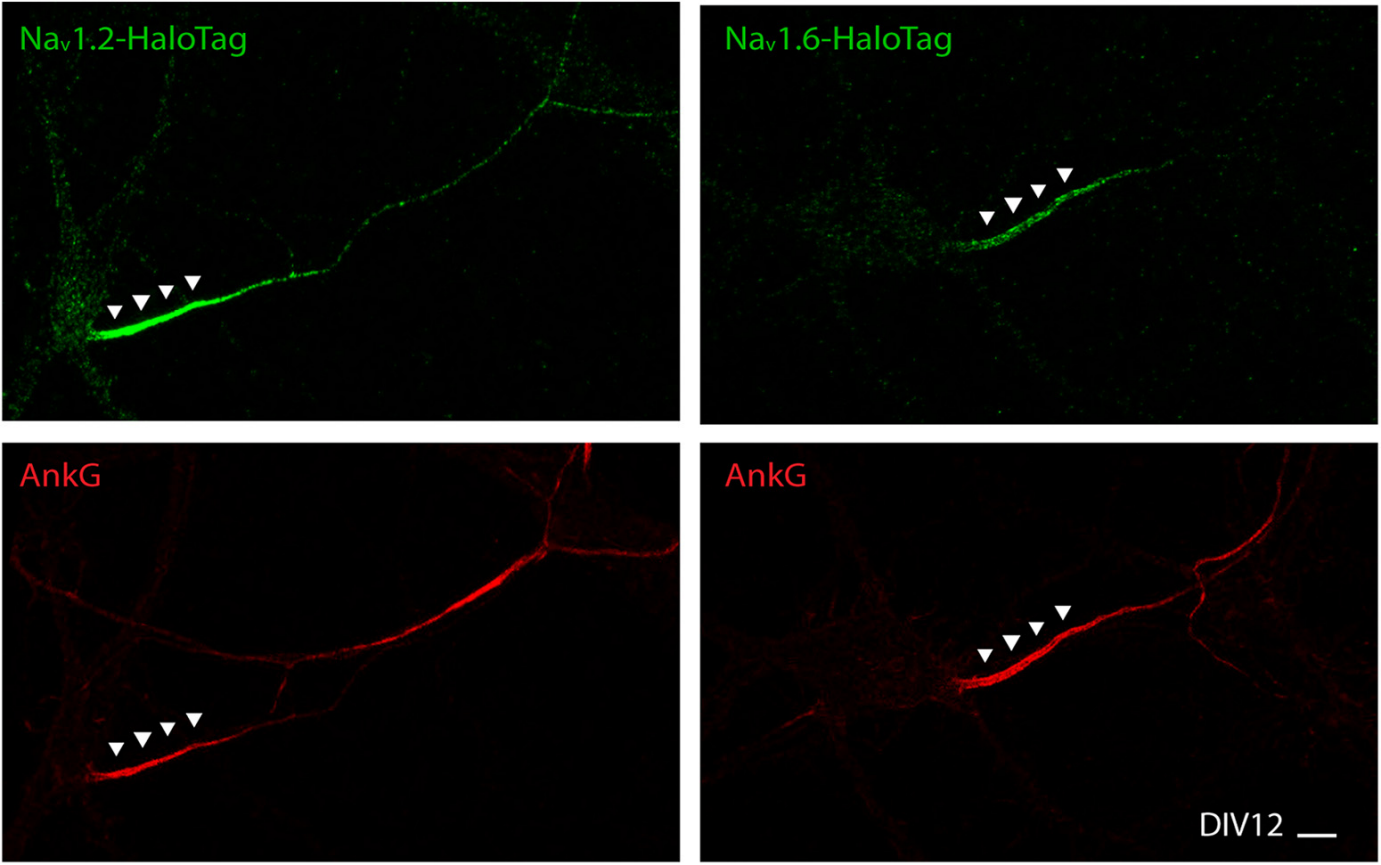
HaloTag-labeled Na_v_1.2 and Na_v_1.6 were enriched in the AIS. With JF646-HTL bulk labeling, Na_v_1.2 (left) and Na_v_1.6 (right) with HaloTag knock-in showed similar distribution patterns as V5 or HA tag knock-in. Scale bar, 10 µm.

**Figure 13.**
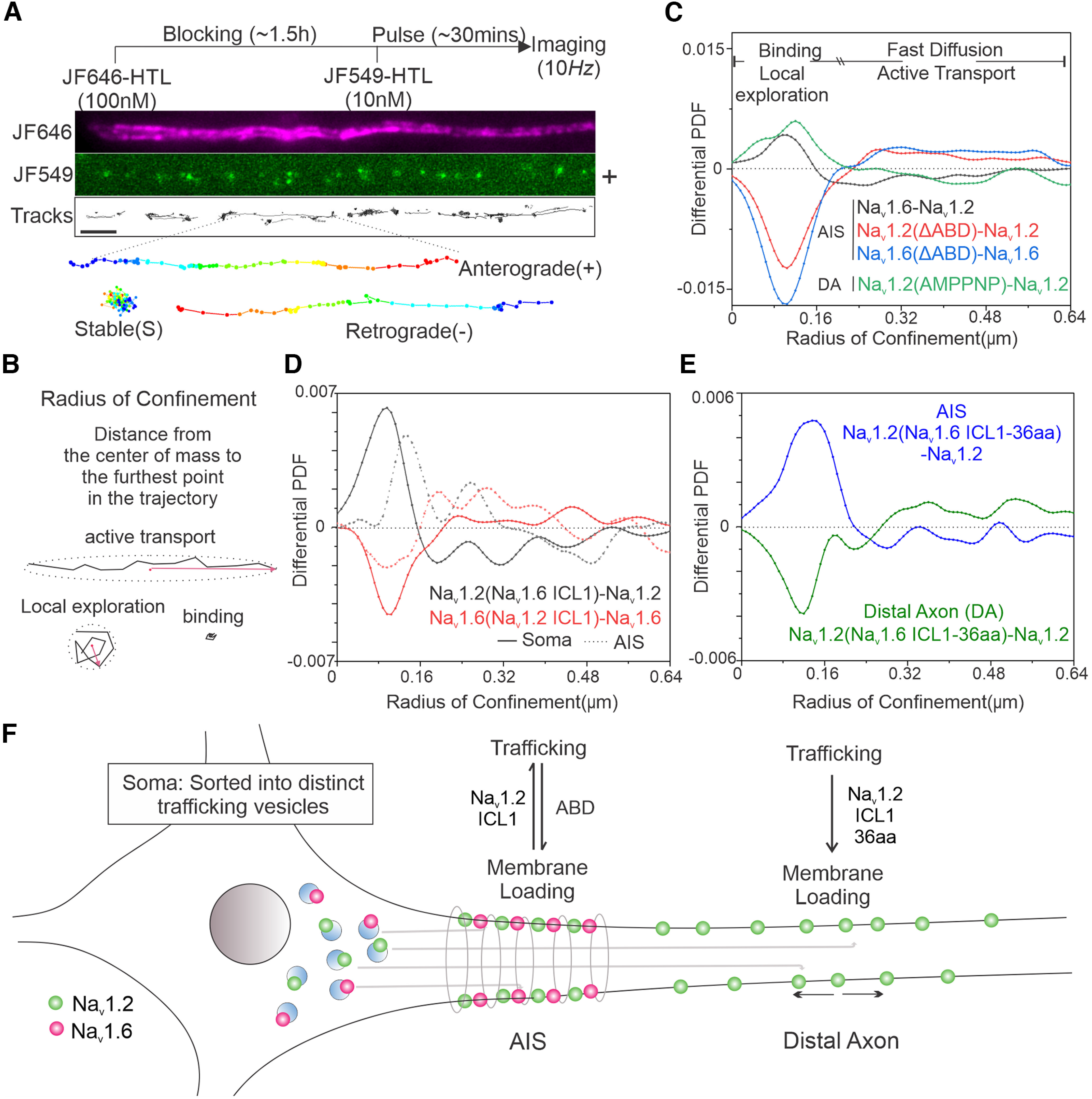
ICL1 mediates the targeting of Nav1.2 to the distal axon. ***A***, Pulse-chase single-molecule imaging of Na_v_1.2. Top, Experimental flowchart. Middle, Representative AIS image, with JF646 bulk labeling image, JF549 pulse-chased single-molecule signals, and analyzed single-molecule moving trajectories. Bottom, Three different types of trajectories, stable binding, anterograde, and retrograde movement (blue to red color change represents time progression). Scale bar, 1 µm. ***B***, Definition of RC for analyzing single-molecule moving dynamics. Stable binding and local exploration events have smaller RCs, whereas active transport and fast diffusion events should have larger RCs. ***C***, Comparative RC distribution curves of Na_v_1.6–Na_v_1.2 (black curve), Na_v_1.2(ΔABD)–Na_v_1.2 (red curve), Na_v_1.6(ΔABD)–Na_v_1.6 (blue curve) in the AIS region and Na_v_1.2(AMPPNP)–Na_v_1.2 (green curve) in the distal axon. Differential PDF = 0 stands for equal fraction. ***D***, Comparative RC distribution curves of Na_v_1.2(Na_v_1.6 ICL1)–Na_v_1.2 (black curve) and Na_v_1.6(Na_v_1.2 ICL1)–Na_v_1.6 (red curve) in soma (solid line) and AIS (dotted line). ***E***, Comparative RC distribution curves of Na_v_1.2(Na_v_1.6 ICL1-36aa)–Na_v_1.2 in AIS (blue) and distal axon (green). ***F***, A model diagram showing that Na_v_1.2 ICL1 is important for suppressing AIS anchoring and facilitating membrane insertion at the distal axon. Movie 6 shows representative single-molecule imaging movies of HaloTag-labeled Na_v_1.2 and Na_v_1.6.

To explain how differential subcellular localizations of Na_v_1.2 and Na_v_1.6 are dynamically established at the molecular level, we sought to use live-cell single-molecule imaging approaches that we established previously (Chen et al., 2014; Liu et al., 2018). These imaging methods with nanometer scale detection sensitivity and high temporal sampling rates similar to PALM (photoactivated localization microscopy) have been widely adopted to study transcription factor and vesicle dynamics in live cells (Chen et al., 2014; Knight et al., 2015; Li et al., 2016; Chong et al., 2018; Liu et al., 2018). We knocked in HaloTag at the C terminus of these two VGSCs, followed by staining with bright, membrane-permeable Janelia Fluor dyes (Grimm et al., 2015). We found that localization patterns of HaloTag-labeled Na_v_1.2 and Na_v_1.6 in the AIS were similar to these tagged with V5 and HA tag (Fig. 12), suggesting that HaloTag labeling did not significantly perturb the subcellular distribution of Na_v_1.2 and Na_v_1.6. Then, we devised a pulse-chase assay in which we first used high concentrations of JF646-HTL to block pre-existing VGSC-HaloTag molecules on the membrane, and then we pulsed cells with JF549-HTL for short durations to label newly synthesized VGSCs that were undergoing trafficking. This technique allowed us to control labeling density by tuning pulse durations and thus obtain long trajectories of trafficking VGSCs under sparse labeling conditions (Fig. 13*A*, Movie 6).

Movie 6. Related to Figure 13. Live-cell, single-molecule imaging of HaloTag-labeled Na_v_1.2 and Na_v_1.6 trafficking dynamics in cultured hippocampal neurons. Scale bar, 10 µm. 10.1523/JNEUROSCI.0086-22.2022.video.6

To establish a simple and effective method to quantify dynamic states (stable binding, local exploration, diffusion, and active transport) associated with trafficking and membrane loading, we took advantage of the Radius of Confinement (RC) parameter that we used successfully to study binding and diffusion states of diverse transcription factors (Lerner et al., 2020). Specifically, the RC is defined as the distance from the center of mass to the farthest point within the trajectory (Fig. 13*B*). Intuitively, fast diffusion and active transport events along the neurites would correlate with larger RCs compared with bound and local exploration states (Fig. 13*B*,*C*). Indeed, we found that ABD deletion in Na_v_1.2 or Na_v_1.6 led to a dramatic reduction of bound (shorter RCs) fractions and an increase in active transport (longer RCs) fractions in the AIS, consistent with known functions of ABD (Fig. 13*C*). Conversely, inhibiting active transport by ATP analog (AMPPNP) significantly reduced active transport (longer RCs) fractions but increased bound (short RCs) fractions in the distal axon (Fig. 13*C*), confirming the ability of the RC analysis to separate distinct dynamic states.

To dissect the molecular basis underlying each dynamic state, we next coupled the RC analysis with extensive genetic perturbations. First, we found that Na_v_1.2 displayed significantly less binding and more active transport events in AIS than Na_v_1.6 (Fig. 13*C*). Similarly, replacing ICL1 in Na_v_1.6 with Na_v_1.2 ICL1 deceased binding and induced more active transport in the AIS and soma. The opposite is true as Na_v_1.2 with Na_v_1.6 ICL1 showed more binding but less active transport events than Na_v_1.2 (Fig. 13*D*). The remarkable consistency in these results support that Na_v_1.2 ICL1 promotes active transport and suppresses retention in the AIS, counterbalancing the anchoring effect of ABD. Complementary with these results, we found that Na_v_1.2 with the membrane anchoring domain ICL-36aa (AA725-760) replaced with the same region from Na_v_1.6 showed much less binding at the distal axon, suggesting that this domain is critical for membrane anchoring of Na_v_1.2 at this region (Fig. 13*E*), consistent with its ability to target GFP to cell membrane (Fig. 11*D*). Together, these results suggest that localization of Na_v_1.2 to the distal axon requires two distinct functions of ICL1, one for reducing anchoring at the AIS and the other for promoting membrane anchoring at the distal axon (Fig. 13*F*).

## Discussion

Here, we overcame the limitations of traditional immunolabeling methods and characterized detailed Na_v_1.2 and Na_v_1.6 subcellular localizations both *in vitro* and *in vivo*. Specifically, our results suggest that Na_v_1.2 is highly concentrated at the somatodendritic region and the proximal AIS during early development. As mice mature, Na_v_1.6 levels increase accompanied with the reduction of Na_v_1.2 levels at these regions. These complementary imaging data support previous electrophysiology results showing that Na_v_1.2 contributes to membrane excitability at the somatodendritic region (Spratt et al., 2019). Interestingly, the reduction of Na_v_1.2 levels at the proximal AIS is accompanied by a shift of the concentration peak of Na_v_1.6 to this region at P30 and P90. Previous electrophysiology experiments revealed that Na_v_1.6 has a much lower activation threshold and larger persistent currents than Na_v_1.2 (Rush et al., 2005). Together with our data, these results suggested that neurons actively adjust their excitability by fine-tuning the membrane composition and localization of Na_v_1.2 and Na_v_1.6 at different developmental stages.

The most pronounced difference between Na_v_1.2 and Na_v_1.6 localizations that we observed is in the distal axon, where their expression and localization patterns showed intricate relationships with the myelination status of the pyramidal neurons. Specifically, Na_v_1.2 covers the AIS and unmyelinated fragments in the distal axon. As neurons undergo myelination, Na_v_1.2 expression levels decrease, accompanied by the exclusion of Na_v_1.2 from the distal axon, consistent with a previous report (Boiko et al., 2001). By contrast, the axonal localization of Na_v_1.6 is largely restricted to the AIS and nodes of Ranvier. In myelinated neurons, Na_v_1.6 becomes the dominant VGSC in the distal axon as we did not detect substantial enrichment of Na_v_1.2 at nodes of Ranvier, consistent with previous results (Caldwell et al., 2000; Boiko et al., 2001). Na_v_1.2 and Na_v_1.6 share conserved sequence and structure homology especially within their transmembrane domains. Thus, their abilities to establish such complex differential localization patterns are particularly intriguing and point to critical channel-type-specific effects of the less conserved intracellular loops, such as the ICL1 that we investigated here.

Here, by dual labeling and two-color super resolution imaging, we first established that once synthesized, Na_v_1.2 and Na_v_1.6 are sorted into distinct pools of trafficking vesicles. We confirmed that the localization of Na_v_1.2 and Na_v_1.6 to the AIS requires previously identified ABD (Garrido et al., 2003; Lemaillet et al., 2003; Gasser et al., 2012). However, separate signals located in ICL1 are responsible for targeting and membrane loading of Na_v_1.2 at the distal axon. Specifically, Na_v_1.6 with Na_v_1.2 ICL1 gains access to unmyelinated fragments in the distal axon. Na_v_1.2 ICL1 alone targets GFP molecules to cell membrane. By coupling pulse-chase labeling with live-cell, single-molecule imaging, we revealed that Na_v_1.2 ICL1 promotes active transport, suppresses retention in the AIS, and promotes membrane loading at the distal axon. Previous reports showed that on neuronal injury, the large persistent currents of Na_v_1.6 at demyelinated sites trigger reverse action of Na^+^–Ca^2+^ exchanger, leading to Ca^2+^ influx that further damages the axon (Craner et al., 2004; Rush et al., 2005). We speculate that coating an unmyelinated axon with a VGSC that conducts smaller persistent currents such as Na_v_1.2 may help protect against Ca^2+^ induced axonal injury.

Our results demonstrated that the complex localization patterns of VGSCs are established by compartment-specific trafficking and loading mechanisms. For a deeper understanding of the underlying molecular mechanism, it would be critical to identify ICL1 interaction partners and their associated pathways in the future. Nonetheless, the developmental regulation and the differential trafficking mechanisms revealed in the current study advance our understanding of how subcellular composition of Na_v_1.2 and Na_v_1.6 are dynamically regulated, which could be a crucial parameter that contributes to their physiological and pathologic functions in the brain.
